# Comparison of the postoperative effects of the erector spinae plane block and local infiltration analgesia in patients operated with lumbotomy surgery incision: Randomized clinical study

**DOI:** 10.1097/MD.0000000000039054

**Published:** 2024-07-26

**Authors:** Sedat Hakimoğlu, Taner Özdemir, Mehmet Selim Çömez, Senem Urfali, Ekrem Yildirak, Sadik Görür, Selim Turhanoğlu

**Affiliations:** a Department of Anesthesiology and Reanimation, Faculty of Medicine, Hatay Mustafa Kemal University, Hatay, Turkey; b Department of Urology, Faculty of Medicine, Hatay Mustafa Kemal University, Hatay, Turkey.

**Keywords:** erector spinae plane block, flank (lumbotomy) incision, local infiltration analgesia

## Abstract

**Background:**

Our aim was to observe the effects of local infiltration analgesia (LIA) or erector spinae plane block (ESPB) methods, which we applied preemptively in patients who were scheduled for surgery with a lumbotomy surgical incision and on intraoperative remifentanil consumption, and to compare the postoperative numerical rating scale (NRS), morphine demand, consumption, and pain degrees.

**Methods:**

Sixty American Society of Anesthesiologists I to III patients aged 18 to 75 years who were due to be operated on with a lumbotomy surgical incision were included in the study. The present study was conducted via prospective, randomized controlled, double-blind trials. After the induction of standard anesthesia, LIA was applied to 30 patients and ESPB was applied to 30 patients preemptively. The dose of remifentanil consumed in the intraoperative period was measured, and the hemodynamic parameters were measured every 5 minutes. Morphine bolus treatment with the postoperative patient–controlled analgesia and rescue analgesia with paracetamol were planned for the patients. Postoperative morphine and additional analgesia consumption, NRS, hemodynamic parameters, and complications were recorded for 48 hours.

**Results:**

There was no difference between the groups in terms of demographic and hemodynamic data. The mean consumption of remifentanil was measured as 455 ± 165.23 µg in the intraoperative ESPB group and 296.67 ± 110.59 µg in the LIA group, and a statistical difference was observed (*P* = .001). In the postoperative follow-ups, the ESPB group drug consumption and NRS score averages were significantly lower at all times (*P* = .01; patient-controlled analgesia-morphine, 41.93 ± 14.47 mg vs 57.23 ± 15.5 mg and additional analgesic-paracetamol: 2.1 ± 1.06 vs 4.27 ± 1.14 g). The mean duration of additional analgesic intake of the groups was 10.6 ± 8.1 in the LIA group, while it was 19.33 ± 8.87 in the ESPB group, a significant difference. The patient satisfaction questionnaire was also significantly in favor of ESPB (*P* = .05).

**Conclusions:**

In conclusion, it has been shown that the intraoperative LIA method is more effective in terms of remifentanil consumption and in controlling pain in operations performed with a flank incision, but the ESPB method provides longer and more effective pain control in postoperative follow-ups.

## 1. Introduction

The main point in regional anesthesia applications is that while providing analgesia in the pain area, it causes no or minimal damage to other functions. Lumbotomy surgical incision (flank incision) is the most commonly used incision in major urological surgical operations.^[1]^ With this incision, operations such as partial nephrectomy, total nephrectomy, pyeloplasty, pyelolithotomy, and nephrolithotomy are performed. After these surgical procedures, severe incision site pain and visceral pain due to peritoneal tension may develop in the early postoperative period. Because of this pain, patients require strong analgesia.^[2]^ Although opioids provide effective analgesia in the treatment of postoperative pain, they may cause side effects such as sedation, nausea, vomiting, and gastrointestinal ileus. Regional anesthesia methods, which are a branch of multimodal analgesia methods, reduce opioid consumption, and thus, the side effects of opioids are avoided.^[3]^ As a result of this advantage, regional anesthesia methods have come to be more commonly used.

Erector spinae plane (ESP) block (ESPB) is a regional anesthesia and analgesia method, which is used especially in abdominal and thoracic surgeries.^[4,5]^ Local anesthetic drug administration to the wound site is an important part of multimodal analgesia. It can be easily performed under ultrasound guidance in all surgical procedures and is widely used due to its low risk of complication.^[6]^ Application of local infiltration analgesia (LIA) to the wound site has taken its place among the multimodal analgesic approaches for postoperative pain management as the importance of nociceptive afferents has come to be understood.^[7]^

Our first aim in this study was to observe and compare the effects of LIA and ESPB methods applied to the wound site after induction of anesthesia on the intraoperative remifentanil consumption of our patients and to compare their postoperative morphine demand and consumption. Our secondary aim was to measure the postoperative pain levels of our patients using the numerical rating scale (NRS) and to compare the side effects associated with morphine consumption in our patients.

We hypothesize that the ESPB method provides more effective perioperative analgesia than the LIA method.

## 2. Material and methods

In Hatay Mustafa Kemal University Health Application and Research Hospital, an average of 68 lumbotomy surgeries are performed annually. The population of the study consists of patients who underwent 68 lumbotomy surgeries. The sample size was determined as 58, with a 5% margin of error and a 95% confidence interval. A total of 66 patients meeting the criteria of the American Society of Anesthesiologists I to III risk group, aged between 18 and 75 years, with a body mass index below 35 kg/m², and undergoing unilateral open lumbotomy surgery were included in the evaluation. Patients with a history of allergy to the drugs used in the study, active infection in the surgical site, mental defects, major vertebral anomalies, known blood clotting disorders, or those using anticoagulant drugs were excluded from the study. Additionally, 2 patients were excluded from the study: one due to medical treatment leading to opioid tolerance and the other refusing to participate.

**Figure 1.**
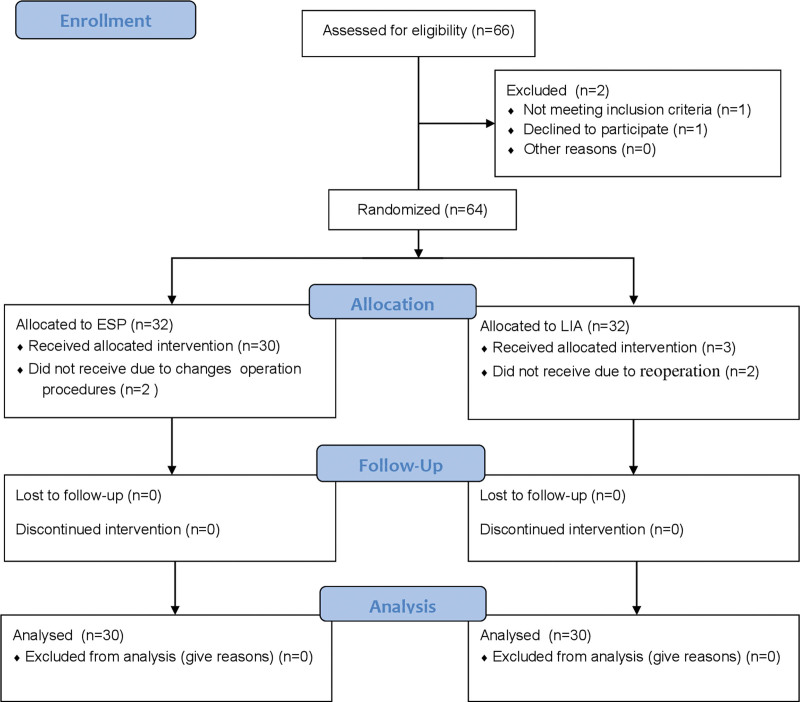
CONSORT flow diagram. CONSORT = Consolidated Standards of Reporting Trials, ESP = erector spinae plane, LIA = local infiltration analgesia.

In the study, a total of 64 patients were enrolled. Initially, it was planned to allocate 32 patients to the ESP group and 32 patients to the LIA group. However, during the course of the study, 4 patients either wished to withdraw or refused intervention, resulting in their exclusion from the study. Therefore, the research was completed with a total of 60 patients. The randomization process and research procedures were illustrated in the Consolidated Standards of Reporting Trials (CONSORT) flow diagram (Fig. 1).

This study was approved by the Hatay Mustafa Kemal University Clinical Research Ethics Committee (protocol number: 2018/12). The study was registered on the Australian New Zealand Clinical Trials Registry (ACTRN12624000415505). Written informed consent was obtained from all participants.

In standard anesthesia induction, patients were preoxygenated with 100% oxygen. Anesthesia induction was achieved via the administration of intravenous (i.v.) propofol (2–3 mg/kg), remifentanil (1 µg/kg), and rocuronium bromide (0.6 mg/kg). To prevent propofol injection pain, i.v. lidocaine (1 mg/kg) was administered prior to propofol and all patients were intubated by the same anesthesiologist. Anesthesia was maintained with a 3-L/min fresh gas flow (50% oxygen and 50% air) using 2% to 2.5 % sevoflurane (1 minimum alveolar concentration mean value). Isotonic 0.9% NaCl was used as the maintenance fluid. The patients were placed in the appropriate surgical lateral decubitus position, and the planned procedures were performed in our study groups. During the surgery, remifentanil was administered at a dose of 0.15 µg/kg/min. Attempts were made to keep the targeted depth of anesthesia in an oscillation range within 20% of the initial systolic arterial pressure (SAP). The depth of anesthesia was tried to be achieved within the targeted SAP range by reducing remifentanil by 25% of the initially calculated infusion dose in cases when SAP decreased below 20% of the initial SAP and by increasing the initial infusion dose of remifentanil by 25% in cases when 20% of the initial SAB was exceeded.

For the anesthesia execution study groups, the patients were randomly assigned to the operating room 30 minutes before the surgery, with the help of a program that generates random numbers. The patient, whose operation was planned with a lumbotomy surgical incision and was stable after anesthesia induction with appropriate hemodynamic parameters, was placed in the 60° lateral decubitus position. Patients were divided into 2 groups: the ESPB group (group ESP, n = 30) and the group in which local anesthetic infiltration was applied to the incision site (group LIA, n = 30). A 20-mL (10 mL of 0.5 mg bupivacaine and 10 mL of 2% lidocaine) local anesthetic was used in each group.

In group LIA, 10 mL of local anesthetic was administered to the skin, superficial subcutaneous, and deep tissue along the incision line before the surgical incision was performed. The surgical procedure was started 15 minutes after the injection. During the operation, 10 mL of local anesthetic was applied again, directly to the subfascial and peritoneal regions.

In group ESP, a linear 38-mm, high-frequency 10- to 15-MHz transducer linear ultrasonography probe was placed to the 2 to 3 cm lateral of the T9 vertebral spinous process on the paramedian sagittal plane. The transverse process of the vertebra, trapezius muscle, erector spinae muscle, and subcutaneous tissue were visualized. The plane between the anterior fascia of the erector spinae muscle and the T9 vertebra transverse process was aimed at from the side using the lumbotomy incision to operate, with a 22-G 80-mm stimuplex needle, using the in-plane technique. The targeted point was reached by advancing the needle in the caudal and cephalic directions at 45° angles; 10 mL of 0.5% bupivacaine and 10 mL of 2% lidocaine local anesthetic were administered in a controlled manner, carrying out negative aspiration at each 5 mL. The procedure was terminated by observing the detached fascial plane after the administration of the local anesthetic drug, and the surgical procedure was started after 15 minutes. The amount of remifentanil used in the intraoperative period was calculated and recorded together with the operation time. Thirty minutes before the completion of the operation, 1-g paracetamol and 1-mg/kg tramadol were administered to the patients.

Postoperative pain management was provided with i.v. morphine using a patient-controlled analgesia (PCA) device. Postoperative morphine consumption was monitored. The PCA device was adjusted to 1-mg bolus dose, 10-minute lock-out interval, and 20-mg 4-hour limit dose. Infusion and loading were not performed.

Paracetamol 1 g was administered i.v. to patients whose NRS values exceed 4 and increase or do not decrease with opioid treatment. Total morphine and paracetamol doses consumed by the postoperative patients at the 0th, 1st, 2nd, 3rd, 4th, 6th, 8th, 12th, 16th, 20th, 24th, 32nd, 40^th^, and 48th hours were recorded. During these follow-ups, the 2 groups were compared via evaluation of the patients’ vital parameters (heart rate, SAP, diastolic arterial pressure, mean arterial pressure, respiratory rate, and SpO2), analgesia requirements, patient satisfaction (0 = poor, 1 = moderate, 2 = good, and 3 = excellent), NRS, and nausea and vomiting scales.

### 2.1. Statistical analysis

The SPSS 20.0 (SPSS Inc., Chicago) statistical package program was used for statistical evaluation. The χ^2^ test was used to compare the categorical variables of the groups. The normal distribution of the numerical parameters of the patients was analyzed using Kolmogorov-Smirnov and histogram tests. Student *t* test was used to compare parameters with normal distribution, and the Mann–Whitney *U* test was used for parameters that did not fit normal distribution; *P* < .05 was regarded as statistically significant.

## 3. Results

Sixty-six patients were included in this study. One patient was excluded from the study because he did not meet the inclusion criteria, and one patient did not want to participate in the study. Sixty-four patients were randomized for the study. Two patients in group ESP were excluded due to changes in operating procedures. Two patients in group LIA were excluded from the study because they underwent reoperation. Therefore, 30 patients in group ESP and 30 patients in group LIA were included in the study (Fig. 1).

The cases in both groups were similar in terms of age, body mass index, and duration of surgery (*P* = .716, *P* = .093, and *P* = .370 respectively). Gender, American Society of Anesthesiologists scores, and type of surgical case were similar in both groups (*P* = .121, *P* = 1.000, and *P* = .428, respectively; Table 1).

**Table 1:** Demographic characteristics (gender, ASA score, surgical case, patient satisfaction).

Variables	Categories		ESPB	LIA	*P*
Gender	Woman	n	18	12	.121*
		%	60.0	40.0	
	Man	n	12	18	
		%	40.0	60.0	
ASA	ASA 1	n	3	3	1.000*
		%	10.0	10.0	
	ASA 2	n	22	22	
		%	73.3	73.3	
	ASA 3	n	5	5	
		%	16.7	16.7	
Surgical case	Pyeloplasty	n	3	2	.428†
		%	10.0	6.7	
	Nephrectomy	n	22	22	
		%	73.3	73.3	
	Hernia	n	2	1	
		%	6.7	3.3	
	Pyelolithotomy	n	3	2	
		%	10.0	6.7	
	Surrenal adenoma	n	0	2	
		%	0.0	6.7	
	Cyst excision	n	0	1	
		%	0.0	3.3	
Patient satisfaction	Poor	n	2	6	.050†
		%	6.7	20.0	
	Moderate	n	6	11	
		%	20.0	36.7	
	Good	n	22	13	
		%	73.3	43.4	

There was no difference between the groups in terms of intraoperative and postoperative hemodynamic data (*P* > .05).

ASA = American Society of Anesthesiologists, ESPB = erector spinae plane block, LIA = local infiltration analgesia.

* Pearson χ^2^.

† Likelihood ratio.

The majority of the patients included in the study were related to nephrectomy (73.3%) cases performed using lumbotomy surgical incision. Nephrectomy cases were equally distributed among the groups, and they were included in the study with the same surgical incision in operations such as pyeloplasty, incisional hernia, pyelolithotomy, adrenal mass, and cyst excision. The distribution of these surgical operations was similar in both groups (*P* = .428; Table 1). In the evaluation of the satisfaction questionnaire answered by the patients at the end of the study, a significant difference was found between the groups in favor of the ESPB (*P* = .05; Table 1); 73.3% of the patients in the ESPB group stated that they were satisfied at the end of the study. This rate remained at 43.4% in the LIA group. There was no statistically significant difference between the groups in terms of intraoperative and postoperative hemodynamic data (*P* > .05).

When the mean values of intraoperative total remifentanil consumption of the study groups were calculated, it was revealed that the average opioid consumption value of the ESPB group was higher than that of the LIA group, and this difference was statistically significant (*P* = .001; Table 2). In cases of longer surgical time, bleeding, volume deficit, etc, which may affect the intraoperative remifentanil consumption of the groups, no significant difference was observed (*P* > .05).

**Table 2:** Intraoperative remifentanil consumption (µg) values.

Total	Mean ± SD	*P*
ESPB	455.00 ± 165.23	.001
LIA	296.67 ± 110.59	

ESPB = erector spinae plane block, LIA = local infiltration analgesia.

**Figure 2.**
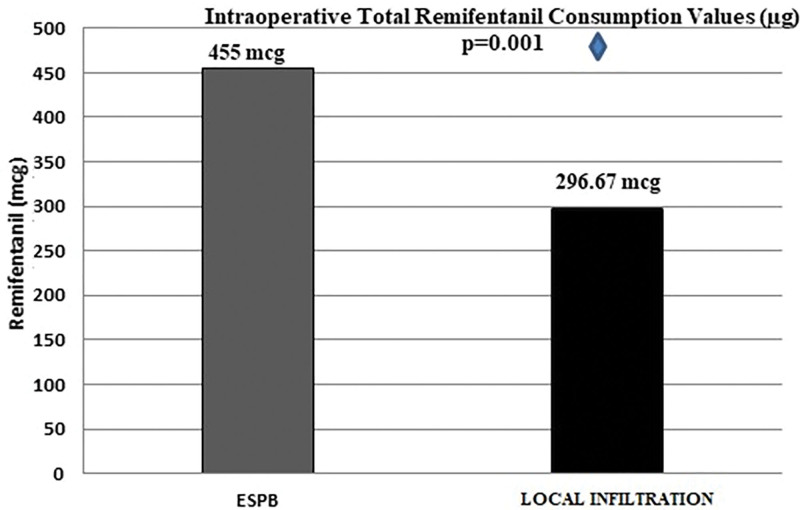
Intraoperative total remifentanil consumption values. ESPB = erector spinae plane block.

The mean total remifentanil consumption in the ESPB group was 455.00 ± 165.23 µg, and the total remifentanil consumption in the LIA group was 296.67 ± 110.59 µg (*P* = .001; Fig. 2).

**Figure 3.**
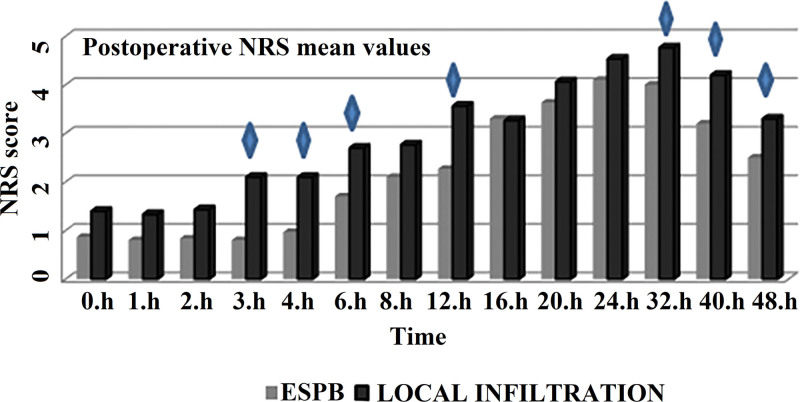
Time-dependent change of numerical rating scale (NRS) levels. ESPB = erector spinae plane block.

When the NRS values of the study groups were examined in the pain assessment scale, it was observed that the mean NRS of the LIA group was higher than that of the ESPB group at all times between the postoperative 0 to 48 hours, but there was a significant difference between the 2 groups only at the 3rd, 4th, 6th, 12th, 32nd, 40th, and 48th hours (*P* = .001, *P* = .003, *P* = .011, *P* = .007, *P* = 0,05, *P* = .013, and *P* = .019, respectively; Table 3). In the graphic view of Table 3, the time periods in which there was a significant difference in the pain score averages between the groups are marked (Fig. 3).

**Table 3:** Postoperative NRS values.

NRS	ESPB	LIA	*P*
	Mean ± SD	Mean ± SD	
0th hour	0.87 ± 1.17	1.40 ± 1.35	.103*
1st hour	0.80 ± 1.16	1.33 ± 1.12	.144*
2nd hour	0.83 ± 1.18	1.43 ± 1.38	.076*
3rd hour	0.80 ± 1.13	2.10 ± 1.71	.001*
4th hour	0.97 ± 1.16	2.10 ± 1.52	.003*
6th hour	1.70 ± 1.74	2.7 ± 1.74	.011*
8th hour	2.10 ± 1.84	2.77 ± 1.57	.137†
12th hour	2.27 ± 1.51	3.57 ± 2.06	.007†
16th hour	3.30 ± 1.93	3.27 ± 1.2	.449*
20th hour	3.63 ± 1.69	4.07 ± 1.48	.136*
24th hour	4.10 ± 1.71	4.53 ± 1.68	.326†
32th hour	4.00 ± 1.49	4.77 ± 1.77	.05†
40th hour	3.20 ± 1.13	4.20 ± 1.73	.013*
48th hour	2.50 ± 1.04	3.30 ± 1.32	.019*

ESPB = erector spinae plane block, LIA = local infiltration analgesia, NRS: numerical rating scale.

* Mann–Whitney *U* test.

† Student *t* test.

In the present study, we also compared and analyzed the total morphine consumption taken with PCA during the postoperative 48 hours and the additional rescue analgesia needed in the case of insufficiency of this treatment.

Additional analgesic consumption time was analyzed in terms of the first hours when the effects of regional anesthesia methods disappeared and our patients started to feel pain. When the average values of drug consumption were calculated, it was found that the consumption averages of the ESPB group were lower than those of the LIA group and this consumption was significant (*P* = .01; Table 4) (Fig. 3). The mean total morphine consumption in the ESPB group was 41.93 ± 14.47 mg, and the total morphine consumption in the LIA group was 57.23 ± 15.5 mg (*P* = .001). Likewise, the mean consumption values of additional analgesics (paracetamol 1 g) by the groups were 2.1 ± 1.06 g in the ESPB group and 4.27 ± 1.14 g in the LIA group (*P* = .001).

**Table 4:** Postoperative opioid and additional analgesic consumption values.

	ESPB	LIA	*P*
	Mean ± SD	Mean ± SD	
Total morphine consumption, mg	41.93 ± 14.47	57.23 ± 15.5	0.001*
Total additional analgesic consumption, g	2.10 ± 1.06	4.27 ± 1.14	0.001†
Additional analgesic consumption time, h	19.33 ± 8.87	10.6 ± 8.10	0.001†

ESPB = erector spinae plane block, LIA = local infiltration analgesia.

* Student *t* test.

† Mann–Whitney *U* test.

**Figure 4.**
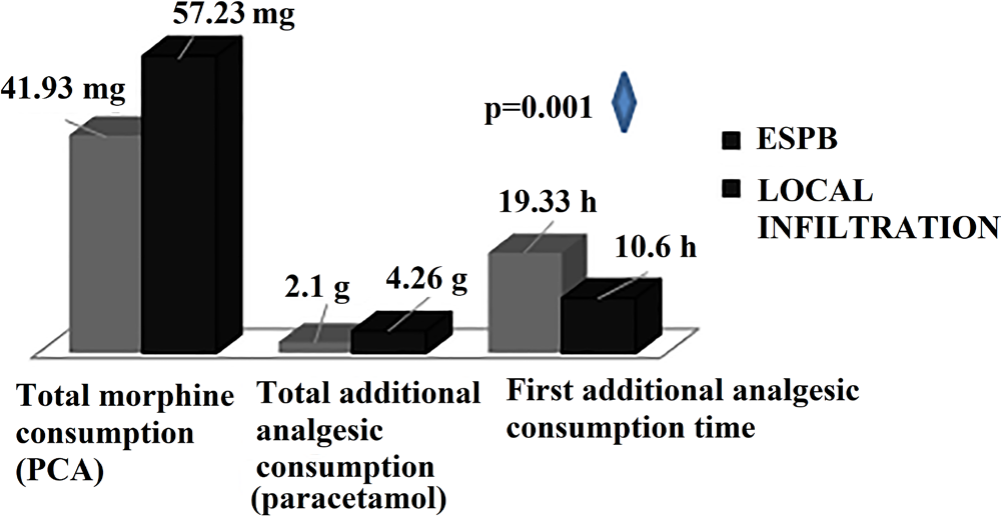
Postoperative total morphine and additional analgesic consumption values. ESPB = erector spinae plane block, PCA = patient-controlled analgesia.

Despite PCA morphine treatment, the additional analgesic requirement time of our patients when NRS was >4 was significant between the groups, and additional analgesia was required earlier in the LIA group (*P* = .001; Table 4; Fig. 4).

When the postoperative nausea-vomiting percentages of the study groups were calculated, it was found that the ESPB group had a lower percentage of nausea-vomiting than the LIA group at all times, but the difference was not significant (*P* = .075).

## 4. Discussion

In this study, we showed that although the contribution of ESPB to intraoperative remifentanil consumption is less than the LIA method, it provides longer and more effective analgesia in the postoperative period. Furthermore, we found that postoperative morphine consumption was lower in patients who were treated with ESPB. Additionally, postoperative additional rescue analgesia was applied later in the group in which we applied ESPB, and it was observed that patient satisfaction was higher in this group to avoid ambiguity.

In recent years, methods that differentially block the dorsal, lateral, and anterior cutaneous nerves of the abdominal and thoracic fascial plane have been preferred since they are less invasive and easier to apply than epidural anesthesia and analgesia. Paraspinal plane blocks (ESP, quadratus lumborum, and paravertebral block), transversus abdominis plane block, or local anesthetic drug infiltration to the wound site have become indispensable techniques of multimodal analgesia in providing regional analgesia in laparoscopic, major urological, and intra-abdominal surgeries of the abdomen.^[8,9]^

Leyva et al used the ESPB in cases of a minimally invasive thoracotomy approach and mitral valve replacement in cardiac surgery.^[10]^ Since cardiac surgery patients are hemodynamically unstable and have perioperative anticoagulation problems, analgesia methods, such as thoracic epidural (hematoma, bleeding, and hypotension) and paravertebral (pneumothorax) block, are hardly possible. ESPB and the insertion of a catheter with it are relatively safe methods in anticoagulated patients due to the distance from vascular structure, the easy application, and the availability of appropriate sonoanatomy.

Another study conducted by Chin et al showed in 3-dimensional computerized tomography images taken after a single level ESPB at the T7 transverse process level that the injected local anesthetic agent proceeded in the caudal-cephalic direction and spread to the paravertebral area in a fresh cadaver. They suggested that caudal-cephalic extension is provided by the thoracolumbar fascia extending throughout the posterior thorax and abdomen and continuing with the nuchal fascia in the neck. This explains how the ESPB can provide an analgesic effect in a wide dermatome area from the C7-T2 level down to the L2-3 level.^[11]^ The interindividual variability in the elongation of thoracolumbar vertebral anatomy explains the interindividual variation in dermatomal spread of the local anesthetic agent. With the paravertebral spread of the local anesthetic agent, the nerve fibers that transmit the sympathetic impulse are also blocked, as are the ventral and dorsal branches of the spinal nerves. Therefore, ESPB can provide visceral analgesia in addition to somatic analgesia.^[11]^ Accordingly, it enables patients to breathe comfortably and helps to reduce opioid consumption, thus avoiding opioid-related respiratory depression.^[12]^ Similarly, local anesthetic is given to this traumatized peritoneal tissue also in the LIA method, and thus, visceral analgesia is achieved.

Having better sonoanatomy compared to other paraspinal plane blocks, being less invasive, and having lower complication risk have made ESPB a more attractive method. Application at a distant area from the wound site and dressing area, compared to local infiltration, is considered another advantage of fascial plane blocks such as ESPB.^[13]^ Although there are randomized studies comparing ESPB and LIA methods in the literature, there is no study comparing these 2 methods under the flank incision in our literature review.^[14]^

Fang et al found no difference in efficacy in a study comparing paravertebral block and ESPB, but they observed significantly less bradycardia, hypotension, and hematoma in the ESP group in terms of complications.^[15]^ They stated that the hypotension and bradycardia experienced in the paravertebral block were due to sympathetic blockade and this block included a high risk of hematoma and pneumothorax due to the proximity to the vascular structure and pleura. In addition, they reported that they achieved a higher success rate with a single injection in the ESPB. It is accepted today that preemptive analgesia methods reduce both postoperative pain scores and perioperative opioid requirements. Therefore, we performed both methods and compared them before the surgical incision.^[16]^

In our study, we preferred remifentanil as an opioid in the intraoperative period due to its advantages, such as short action time and rapid onset and termination of its effect. Thus, we were able to monitor intraoperative opioid consumption more closely. The average dose of remifentanil consumed intraoperatively in both groups remained around 0.15 µcg/kg/min and the hemodynamics remained stable, indicating that both methods contributed to intraoperative analgesia. The reason why the use of intraoperative remifentanil was less in the LIA group may be the anesthesia that it creates in the surgical incision area. In the LIA group, a local anesthetic applied to the incision line before the surgical incision may temporarily block the nociceptive stimulus in the area and prevent the release of pain-related mediators. Since the target in ESPB is the paraspinal fascial plane, the block onset time is longer than in LIA. This mechanism suggests that LIA reduces intraoperative opioid consumption compared to ESPB. The shorter duration of its effect compared to the ESPB suggests that absorption from the surgical incision is high.

We observed that the analgesia quality of both groups was statistically effective and similar in the first 6 hours, and the analgesia quality of the ESPB group was more effective than the LIA group in the following hours. The increase in the mean duration of the block effect of the ESPB group (19.33 ± 8.87 hours) in our patients to almost twice that of the LIA group (10.6 ± 8.1 hours) suggests that the local anesthetic administered remained only in the fascial plane, and the elimination time of the local anesthetic was longer.

Regional methods reduce postoperative opioid consumption and minimize possible complications such as nausea, vomiting, and itching.^[17,18]^ Reducing the incidence of opioid-related nausea and vomiting by reducing opioid consumption is also very important in other surgeries of the gastrointestinal system, especially esophageal surgery.^[14]^ In our study, it was revealed that the ESPB group had a lower rate of nausea and vomiting in the postoperative follow-up than the LIA group. The complaints of nausea in the later hours (16–48 hours) in our cases are suggested to be related to PCA and cumulative morphine consumption.

The current study has some limitations. Since the ESPB was first performed after the induction of general anesthesia, the success and sensory evaluation of the block could not be made. Second, the sample size of the study was not sufficient for the occurrence of ESPB complications. Finally, a volume-response or concentration-response study was not performed on the optimal volume and concentration of a local anesthetic drug used in ESPB, which is performed in order to obtain a better analgesic effect and fewer side effects in patients operated on with a flank incision.

## 5. Conclusion

In conclusion, the intraoperative LIA method was effective in controlling pain in operations performed with a flank incision in terms of opioid consumption; however, the ESPB method provided longer and more effective pain control in postoperative follow-ups. We believe that there is a need for future studies with a larger patient population, for multicentered randomized controlled studies, and even for studies that combine the use of LIA and ESPB.

## Acknowledgements

The authors thank the patients and controls for their voluntary support for this study.

## Author contributions

**Investigation:** Sedat Hakimoğlu, Taner Özdemir, Mehmet Selim Çömez, Ekrem Yildirak, Selim Turhanoğlu

**Methodology:** Sedat Hakimoğlu, Sadik Görür, Selim Turhanoğlu

**Writing – original draft:** Sedat Hakimoğlu, Taner Özdemir

**Writing – review & editing:** Sedat Hakimoğlu, Sadik Görür, Selim Turhanoğlu

**Data curation:** Taner Özdemir, Mehmet Selim Çömez, Senem Urfali, Ekrem Yildirak, Sadik Görür

**Resources:** Taner Özdemir, Senem Urfali

**Software:** Mehmet Selim Çömez

**Visualization:** Senem Urfali
